# Cultivation of Cells in a Physiological Plasmax Medium Increases Mitochondrial Respiratory Capacity and Reduces Replication Levels of RNA Viruses

**DOI:** 10.3390/antiox11010097

**Published:** 2021-12-30

**Authors:** Michail V. Golikov, Inna L. Karpenko, Anastasiya V. Lipatova, Olga N. Ivanova, Irina T. Fedyakina, Viktor F. Larichev, Natalia F. Zakirova, Olga G. Leonova, Vladimir I. Popenko, Birke Bartosch, Sergey N. Kochetkov, Olga A. Smirnova, Alexander V. Ivanov

**Affiliations:** 1 Engelhardt Institute of Molecular Biology, Russian Academy of Sciences, 119991 Moscow, Russia; cool.mik3492594@yandex.ru (M.V.G.); ilkzkil@gmail.com (I.L.K.); lipatovaanv@gmail.com (A.V.L.); olgaum@yandex.ru (O.N.I.); nat_zakirova@mail.ru (N.F.Z.); leonova-kozma@mail.ru (O.G.L.); popenko@eimb.ru (V.I.P.); snk1952@gmail.com (S.N.K.); 2 Gamaleya National Research Centre for Epidemiology and Microbiology of the Ministry of Russia, 132098 Moscow, Russia; irfed2@mail.ru (I.T.F.); vlaritchev@mail.ru (V.F.L.); 3 Inserm U1052, Cancer Research Center Lyon, University of Lyon, 69000 Lyon, France; birke.bartosch@inserm.fr

**Keywords:** culture medium, Plasmax, serine biosynthesis, respiration, glycolysis, mitochondrial network, hepatitis C virus, influenza virus, SARS-CoV-2, reactive oxygen species

## Abstract

Changes in metabolic pathways are often associated with the development of various pathologies including cancer, inflammatory diseases, obesity and metabolic syndrome. Identification of the particular metabolic events that are dysregulated may yield strategies for pharmacologic intervention. However, such studies are hampered by the use of classic cell media that do not reflect the metabolite composition that exists in blood plasma and which cause non-physiological adaptations in cultured cells. In recent years two groups presented media that aim to reflect the composition of human plasma, namely human plasma-like medium (HPLM) and Plasmax. Here we describe that, in four different mammalian cell lines, Plasmax enhances mitochondrial respiration. This is associated with the formation of vast mitochondrial networks and enhanced production of reactive oxygen species (ROS). Interestingly, cells cultivated in Plasmax displayed significantly less lysosomes than when any standard media were used. Finally, cells cultivated in Plasmax support replication of various RNA viruses, such as hepatitis C virus (HCV) influenza A virus (IAV), severe acute respiratory syndrome-related coronavirus 2 (SARS-CoV-2) and several others, albeit at lower levels and with delayed kinetics. In conclusion, studies of metabolism in the context of viral infections, especially those concerning mitochondria, lysosomes, or redox systems, should be performed in Plasmax medium.

## 1. Introduction

Viruses represent a vast group of etiologic agents of acute or chronic human diseases. They have attracted much attention from both scientists and society in recent decades, especially since the discovery of human immunodeficiency virus (HIV), hepatitis C virus (HCV), and of course after identification of severe acute respiratory syndrome-related coronavirus 2 (SARS-CoV-2) which started a pandemic in early 2020. Extensive research has characterized the life cycles of these viruses and proposed drugs that can either cure the infection (as in the case of HCV) [1] or suppress active replication (as in case of HIV or hepatitis B virus) [2,3]. However, study of pathogenic events associated with these viral infections is still ongoing, as current in vitro models do not mimic the processes induced by these viruses in the infected cell/tissue in vivo.

Numerous lines of evidence show that cell proliferation, (de)differentiation, or activation is associated with remodeling of metabolism: the shift between glycolysis and mitochondrial respiration as well as with changes in other catabolic/anabolic pathways and redox systems [4,5,6,7,8]. In recent years, it has become clear that the investigation of cellular metabolism is affected by cell cultivation conditions. First, the environment which cells find in monolayers in 2D culture is quite different from that in tissue or 3D cultures [9]. Second, our normoxic condition (21% oxygen) is, rather, superoxic, as in most tissues— except the lung—the oxygen levels are 3.4–6.8% [10,11]. Third, widely spread DMEM and many other common media contain elevated levels of glucose, thus imposing a “glycemic” insult to cells [12,13]. Levels of many other metabolites are also far from those found in physiological cell settings [14]. Finally, such media often lack many metabolites that cells take up from blood (for example, [15]), e.g., the widespread supplementation of media with pyruvate in the absence of lactate, present in blood at millimolar concentrations [16], that shifts the redox state to reductive [17]. Taking all this into account, several laboratories have proposed culture media recipes to overcome these drawbacks. Sabatini’s and Tardito’s groups presented human plasma-like medium (HPLM) [18] and Plasmax [19], respectively, whose composition reflects plasma in terms of major components (concentrations > 2 µM). They contain a reduced amount of glycose, pyruvate and arginine compared to standard media and are supplemented with a variety of components that are present in plasma but are omitted from DMEM and other media. It has been shown that use of such media normalizes the urea cycle, glutaminolysis and hypoxia-inducible factor (HIF) signaling and reprograms serine biosynthesis [19].

As HPLM and Plasmax have only recently been described, their impact on other cellular metabolic pathways remains obscure. In addition, there are no data on the investigation of virus replication and associated metabolic changes using these culture media. So the goal of this study was to access the effect of Plasmax medium on cellular respiration and glycolysis, and its impact on replication of various RNA viruses including HCV, influenza A virus, SARS-CoV-2, and several enteroviruses.

## 2. Materials and Methods

### 2.1. Cell Lines and Their Cultivation

Human hepatoma Huh7.5 cells were a kind gift from Prof. Charles Rice (Rockefeller University, NY, USA). Human adenocarcinoma A549 (CCL-185) and cervical adenocarcinoma HeLa (CCL-2) cell lines were obtained from ATCC (USA). The Vero E6 cells derived from green monkey kidney were from the Russian National Collection of Cell Cultures at the National Research Center for Epidemiology and Microbiology, named after Honorary Academician N.F. Gamaleya of the Ministry of Health of the Russian Federation (Moscow, Russia). The cells were checked for mycoplasma contamination by standard PCR every two weeks. All cells were cultivated at 37 °C in a humid atmosphere containing 5% CO_2_. The standard media were supplemented with 10% fetal bovine serum (FBS) and 2 mM glutamine if not stated otherwise. As a standard medium, we used DMEM for Huh7.5 cells, DMEM-F12 for A549 cells, MEM for HeLa, and DMEM supplemented with 2% FBS for Vero E6 cells.

Plasmax medium was assembled as described by Vande Voorde et al. [19]. The composition of the medium and catalogue numbers of its components are presented in Supplementary Table S1. All stock solutions were stored at −86 °C and the medium was assembled prior to the experiments, filtered through 0.22 µm polyethylene sulfone membrane and stored at +4 °C.

### 2.2. Cell Morphology Analysis

Twenty-four hours prior to analysis cells were seeded on 6-well-plates at 2 × 10^5^ cell/well density. Morphology was visualized on Olympus IX83 microscope with a differential interference contrast (DIC) or phase contrast at 20x/0.75 objective, and images were captured using CellSence software.

### 2.3. Real-Time—Quantitative PCR (RT-qPCR)

Cells were seeded onto 6-well plates in a given medium and harvested at 95% monolayer density by scraping. RT-qPCR was performed in accordance with [20,21]. Briefly, total RNA was purified using a High Pure RNA Isolation Kit (Roche Life Sciences, Basel, Switzerland) according to the manufacturer’s instructions, and reverse transcribed with random hexamer primer and RevertAid enzyme (Thermo Fisher Scientific, Waltham, MA, USA). PCR was performed using primers listed in Table S2. A standard reaction mixture (10 μL) contained the respective primers, cDNA equivalent to 50 ng total RNA, and qPCRmix-HS SYBR (Evrogen). The real-time PCR thermal conditions were 55 °C for 5 min, 95 °C for 10 min, followed by 40 cycles each at 95 °C for 10 s, and 57 °C for 1 min (signal collection temperature). The results were analyzed by the ΔΔCt approach.

### 2.4. Toxicity of Metabolic Inhibitors

Cells were seeded onto 96-well plates at a density of 1.8 × 10^4^ cells/well. Eighteen hours later the inhibitors were added, and the cells were kept for an additional 72 h. For the compounds dissolved in DMSO, the solvent was added to the control wells as a vehicle. Then, the medium was changed with a fresh medium containing 500 µg/mL 3-[4,5-dimethylthiazol-2-yl]-2,5 diphenyl tetrazolium bromide (MTT), after 4 h incubation at 37 °C the media was removed, and formazan crystals were dissolved in 2-propanol supplemented with 0.04 M HCl, and optical absorbance was measured at 544 nm with a background at 620 nm on a Chameleon V microplate reader (Hydex Oy, Turku, Finland). The values were normalized to the absorbance for the control cells in the absence of a tested compound.

### 2.5. Measurement of Glycolysis and Mitochondrial Mespiration

Glycolysis and mitochondrial respiration were assessed by Seahorse technology (Agilent Technologies, Santa-Clara, CA, USA) on a XFe24 analyzer according to manufacturer’s instructions with slight modifications. Briefly, 24 h prior to analysis the cells were seeded via XF24 Cell Culture Microplate into (1.5 × 10^5^ cells/well) in standard media or Plasmax with four replicates. For the MitoStress test, 45 min before analysis the media was changed to DMEM or Plasmax, lacking phenol red dye and bicarbonate and supplemented with 25 mM (conventional medium) or 5.5 mM (Plasmax) glucose, 2 mM pyruvate and 2 mM glutamine, and the plate was kept at 37 °C at normal atmosphere. To evaluate respiration-linked ATP production, maximum respiratory capacity, and non-mitochondrial respiration, ATP-synthase inhibitor oligomycin, uncoupler FCCP, and a mixture of complex I and III inhibitors rotenone and antimycin were added to final concentrations of 1 µM (Oligomycin), 0.45 µM (FCCP), and 1 µM each (rotenone/antimycin). For each condition, three readings were performed at 3 min intervals.

In case of the GlycoStress test, 30 min prior to analysis, the medium was changed to DMEM or Plasmax lacking phenol red dye, bicarbonate and glucose and supplemented with 2 mM pyruvate and 2 mM glutamine. During the assay 5.5 and 25 mM Glycose (DMEM), 1 µM oligomycin and 50 mM 2-deoxyglucose were added to access basal glycolysis, maximal glycolytic capacity, and non-glycolytic acidification, respectively.

The raw data were processed by Seahorse Wave Desktop software (Agilent Technologies) and further analyzed by GraphPad Prism (GraphPad Software, La Jolla, CA, USA).

### 2.6. Quantification of Mitochondrial and Lysosomal Mass

Lyophilized Mitotracker Red CM-H2XROS and LysoTracker Red DND-99 (Thermo Fisher Scientific, USA) were dissolved in DMSO at 1 mM concentration. The cells were seeded on 12-well plates at 2 × 10^5^ cell/well density. When culture density reached 75–85%, medium was removed, and 300 µL of pre-heated medium containing 1.5 µM of the respective dye was added. After 40–45 min incubation at 37 °C the cells were washed with warm medium, harvested with 0.05% trypsin-EDTA solution and subsequent centrifugation and resuspended in a warm culture medium lacking phenol red. The fluorescence intensity was measured using BD LSR Fortessa Flow Cytometer (Becton Dickinson, Franklin Lakes, NJ, USA) at 610 nm after excitation with 561 nm laser. Data analyses were performed using the Flowing Software (Turku Centre for Biotechnology, Finland).

### 2.7. Visualization of Mitochondria by Confocal Microscopy

The cells were seeded in a respective media into 35-mm confocal dishes with glass inserts. Twenty four hours later Mitotracker Red CM-H2XROS was added to 1.5 µM final concentration in a fresh pre-heated medium, and 45 min later the cells were washed with warm PBS and then with phenol red-free medium. Confocal 8-bit digital images were acquired using a Leica TCS SP5 confocal laser-scanning microscope (Leica Microsystems, Wetzlar, Germany) equipped with a HCX PLAPO CS 63×1.4 oil immersion lens. Fluorescence was excited at 543 nm and registered in the 560–600 nm range. Mitochondrial morphology phenotype was accessed in at least 40 cells per condition. Each cell was classified according to general status of the mitochondria phenotype. A cell was assigned to filamentous phenotype if >80% mitochondria in it were elongated, to fragmented phenotype if <50% mitochondria were tubular, or to intermediate phenotype. Scoring was performed blind, independently by two people. Percentage of cells with these phenotypes were evaluated in 3 or 5 independent experiments that were defined as separate experiments with similar culture conditions occurring on different days from other replicates.

### 2.8. Evaluation of Mitochondrial Turnover Using Mitotimer

HeLa cells on a 6-well plate were transfected with the plasmid encoding MitoTimer protein (plasmid #52659, Addgene) [22]. Briefly, 4 µg plasmid was mixed with 12 µL Turbofect reagent (Thermo Scientific) in 200 µL OPTI-MEM, and after 30 min incubation at room temperature added to the cells at 50% monolayer. Twenty-four hours post-transfection the cells were seeded onto 6-cm dishes. Upon 90% monolayer, the cells were collected by trypsin-EDTA treatment with consequent centrifugation and resuspended in 1mL preheated PBS. The cells expressing the fluorescent protein were selected on a BD Aria III cell sorter (Becton Dickinson) and collected into fresh pre-heated media supplemented with penicillin-streptomycin. On the following day the medium in some samples was replaced with Plasmax, and after 7 days the cells were subjected to Fluorescence activated cell sorting (FACS) analysis by measuring levels of green and red fluorescence at 488/530 nm (green channel) and 561/610 nm (red channel) or confocal microscopy.

### 2.9. Infectious Studies

#### 2.9.1. Hepatitis C Virus (HCVcc)

Huh7.5 cells were seeded on 6-well plates at a density of 3 × 10^5^ cells/well in DMEM-F12 medium and 24 h later inoculated with HCV at 0.1 multiplicity of infection (MOI). Four hours post-infection the medium was removed, the cells were washed with PBS (3 × 1 mL) and the fresh medium was added. The cells were harvested 6 days later. RNA was purified, and genomic HCV RNA levels were quantified as described previously [21].

#### 2.9.2. HCV Sub-Genomic Replicon

Huh7.5 virion stock was obtained according to the standard protocol [23]. A stable Huh7.5 cell line harboring sub-genomic HCV RNA corresponding to the HCV JFH-1 strain was described previously [21]. The cells were cultivated in standard DMEM medium supplemented with 400 μg/mL G418 prior to being seeded for the experiment. For analysis of virus replication, the cells were seeded onto 6-well plates at a density of 2 × 10^5^ cells/well, and after attachment of cells the medium was replaced by fresh DMEM, Plasmax, Plasmax supplemented with glucose to the level of 4.5 g/L or DMEM with 2.5% FBS. The cells were harvested 2, 4, 6, 8, and 10 days after medium change. RNA was purified, and genomic HCV RNA levels were quantified as described in [21].

#### 2.9.3. Influenza A Virus (IAV)

Influenza A virus of A/California/7/2009 (H1N1)pdm09 strain was from the Russian Federation National Virus Collection at the National Research Center for Epidemiology and Microbiology named after Honorary Academician N.F. Gamaleya of the Ministry of Health of the Russian Federation. A549 cells were seeded 1–2 days prior to infection at 4 × 10^4^ cells per well in 6-well plates in complete DMEM-F12/Plasmax medium. When cells reached 95–100% confluence, IAV was added at 0.1 MOI in DMEM-F12/Plasmax Media supplemented with 2.5% FBS and TPCK-treated trypsin (Thermo Fisher Scientific) at a final concentration of 2 μg/mL (IAV infection media). The cells were shaken manually every 20 min, and two h post-infection the media was changed with fresh DMEM-F12/Plasmax media supplemented with 2.5% FBS. Total RNA was purified from conditioned medium using High Pure RNA Isolation Kit (Roche Life Sciences, Switzerland) according to the manufacturer’s instructions, and the levels of viral RNA were quantified by RT-qPCR as described in Section 2.3 using primers listed in Table S2.

#### 2.9.4. SARS-CoV-2

The study used the SARS-CoV-2 human coronavirus, passage 3, with infectivity of 10^7.5^ TCID_50_/_mL_. Strain description: hCoV-19/Russia/Moscow-PMVL-12/2020 (EPI_ISL_572398) GISAD: PMVL-12. Booking reference EPL_ISL_572398. Vero E6 cells were seeded 1-2 days prior to infection at 4 × 10^4^ cells per well in 6-well plates on complete DMEM/Plasmax medium. When the cells reached 95–100% confluency, SARS-CoV2 viral stocks were diluted to final concentration 0.1 MOI in DMEM/Plasmax media supplemented with 2.5% FBS. The cells were inoculated for 2 h with this viral dilution with manual shaking every 20 min followed by substitution of culture medium with a fresh DMEM or Plasmax medium supplemented with 2.5%/ FBS. Quantification of viral RNA in conditioned medium was performed using specific primers (Table S2) as described for influenza virus.

#### 2.9.5. Other Viruses

The following viral strains were used: vesicular stomatitis virus Indiana strain (VSV-I), Sabin vaccine strains of type 1 and 3 poliovirus (PV3), Newcastle disease virus strain H2 (NDV-H2), and Coxsackie B5 virus strain (CVB5) LEV14 (GenBank: MG642820.1). The enteroviruses were propagated in RD cells, VSV-I in BHK21 cells, NDV-H2 in Vero E6 cells. RD, BHK21 and Vero cells were cultured on DMEM (Gibco) media, containing 10% FBS and penicillin/streptomycin in standard concentrations. Virus titers (such as TCID50/mL) were measured according to the standard protocol [24]. Titration-based measurements of virus production were performed in four biological replicates. The cells were seeded in 12-well plates at a density of 3 × 10^5^ cells per well and later infected: with different MOI (0.01 for VSV-1, 0.1 for CVB5, PV1, and PV3 enteroviruses, 0.5 for NDV). Levels of infection were accessed by quantification of cytopathogenic effect: cell viability was quantified by MTT test as described above.

### 2.10. Measurement of ROS Production

Production of reactive oxygen species was accessed using redox-sensitive fluorescent probes: 2′,7′-dichlorodihydrofluoresceine diacetate (DCFH2DA), dihydroethidium (DHE), and MitoSOX. The cells were stained as described earlier [20]. In case of uninfected cells fluorescence intensity was measured by flow cytometry at 488/530 nm in case of DCFH2DA or 561/610 nm in case of DHE and MitoSOX using BD LSR Fortessa Cytometer. Mean levels of fluorescence were registered for viable cells.

In case of infections the fluorescence was measured on a microplate reader Chameleon V at 488/535 nm for DCFH2DA or 510/590 nm in case of DHE and MitoSOX.

### 2.11. Statistical Analysis

All independent experiments were performed in triplicate, and at least three independent experiments were carried out. Statistical analysis was performed with GraphPad Prism software (GraphPad Software Inc.). All data are presented as mean ± SD. Differences between two groups were analyzed using the two-tailed unpaired Student’s *t*-test, whereas multiple comparisons were performed by Analysis of variance (ANOVA) with Tukey’s post-hoc test. *p*-values < 0.05 were considered statistically significant if not stated otherwise.

## 3. Results

### 3.1. Cells Grown in Plasmax Medium Exhibit Different Morphology and Proliferation Rates Compared to Culture in Classical Medium

**Figure 1:**
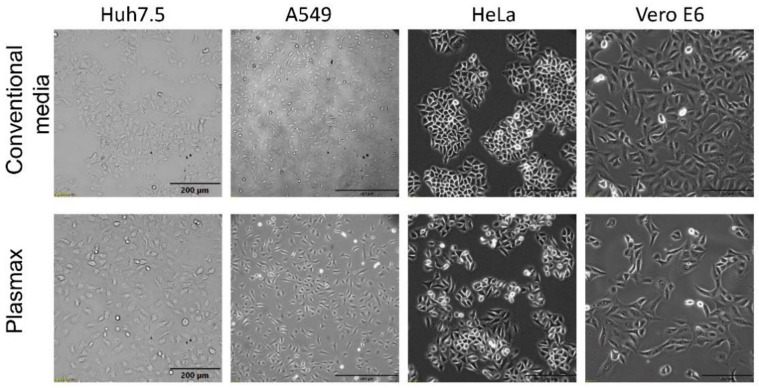
Cultivation of cells in Plasmax medium affects cell morphology of various cell lines. The cells were cultivated in Plasmax or respective conventional media for 7 days, and images were captured using a phase-contrast microscope/using phase-contrast microscopy. The bars denote 200 µm with the exception of A549 cells, for which it denotes 500 µm.

The initial experiments were performed with two cell lines: human hepatoma Huh7.5 and lung adenocarcinoma A549 cells maintained in high-glucose DMEM and DMEM-F12 media supplemented with 10% FBS, respectively. These media were replaced or not with Plasmax, and both cell lines were maintained for ten days with passaging at 90% confluence. Media were replaced whenever cells were split. Starting from day 3 after medium change into Plasmax, Huh7.5 cells changed morphology by becoming more contrasted and homogenous within a population (Figure 1). Moreover, they formed less tight cell-cell contacts, as more individual cells were seen. For A549 cells no notable change in morphology was seen.

To unveil possible changes in cell metabolism in response to Plasmax, expression levels of metabolic enzymes from various pathways were quantified by real-time-RT-PCR. Upregulated expression of genes that encode enzymes of de novo serine biosynthesis (PSPH, PSAT, PHGDH) and asparagine synthetase (ASNS) (Figure S1) were detected in Huh7.5 cells Since these enzymes are known to be controlled by the ATF4 transcription factor of integrated stress response [25,26], this effect may not be due to metabolic adaptation to Plasmax but merely due to exhaustion of some nutrients in the medium. Indeed, induction of all these genes disappeared when medium was replaced every 24 h (Figure S1). So in all the following experiments, Plasmax medium was refreshed every day to avoid artifacts.

Modification of the cultivation protocol did not prevent changes in the morphology of Huh7.5 cells. Next, two additional cell lines were added to our studies: human cervical adenocarcinoma HeLa and African green monkey kidney Vero E6 cells. HeLa cells are normally propagated in MEM supplemented with 10% FBS, and Vero E6 in DMEM supplemented with 2% FBS. HeLa cells in Plasmax exhibited less tight cell–cell contacts, in contrast to MEM medium where individual cells were seen (Figure 1). Vero E6 became more elongated and flattened in Plasmax.

### 3.2. Plasmax Medium Affects Mitochondrial Activity

**Figure 2:**
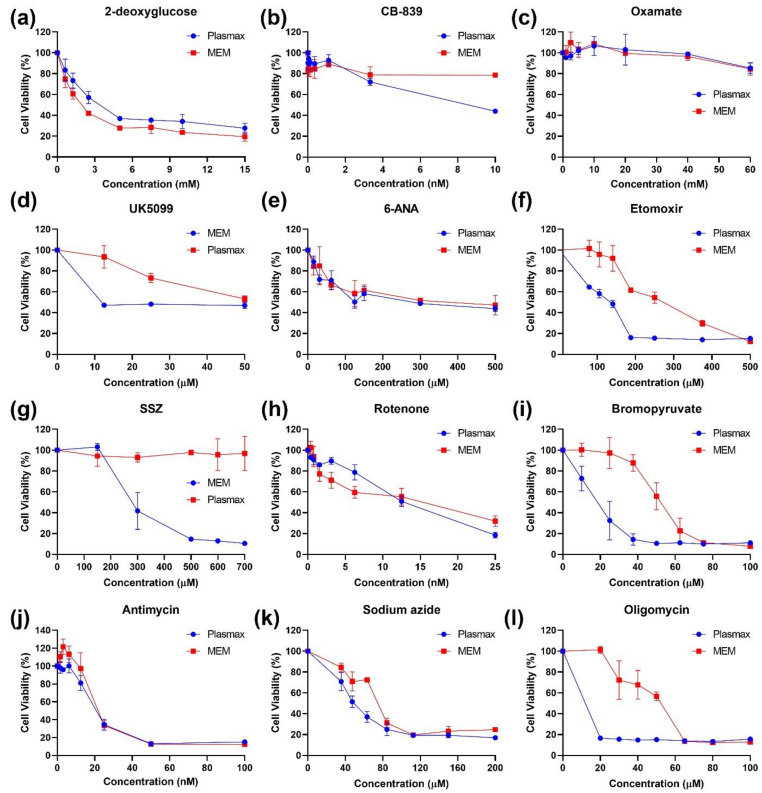
Cultivation of HeLa cells in Plasmax leads to their enhanced sensitivity to inhibitors of fatty acid catabolism, mitochondrial respiratory complexes, and to a ferroptosis inducer. The HeLa cells cultivated in Plasmax or MEM as a conventional medium were treated with inhibitors of glycolysis (2-deoxyglucose and oxamat—**a**,**c**), glutaminolysis (CB-839—**b**), mitochondrial pyruvate antiporter (UK5099—**d**), pentose phosphate pathway (6-ANA—**e**), fatty acid degradation (etomoxir—**f**), cysteine/glutamate antiporter (sulfasalazine, SSZ—**g**), or of respiratory complexes I-V (rotenone, bromopyruvate, antimycin, sodium azide, and oligomycin—**h**–**l**) for 72 h, and cell viability was accessed by conventional MTT test. The values were normalized to those of the untreated cells.

The next step was to access the influence of Plasmax medium on various metabolic pathways. As tools, a series of pharmacologic inhibitors of various metabolic enzymes were employed. Most were evaluated in HeLa cultures (Figure 2 and Figure S2), and the key results were verified in Huh7.5 and A549 cells (Figures S3 and S4).

Briefly, inhibitors of glycolysis (2-deoxyglucose, oxamate), pentose phosphate pathway (6-ANA), and gluta-minolysis (CB-839) and other pathways were found equally active in cells maintained in Plasmax and the respective classical medium (Figure 2a–c,e). The first difference was noted for UK5099 that blocks the activity of mitochondrial pyruvate transporter (MPC): cells in Plasmax were more susceptible to the compound (Figure 2d). Second, sulfasalazine, an inhibitor of the glutamate/cistine antiporter (xCT) and an inducer or ferroptosis [27], was non-toxic for cells in Plasmax (Figure 2g). However, the difference was explained by the presence of selenite in Plasmax as, in the case of Huh7.5 cells, its addition to DMEM at a similar level completely prevented cell death (Figure S3).

The third difference was found for etomoxir, an inhibitor of carnitine palmitoyltransferase-1 (CPT-1) that blocks transport of fatty acyl chains into mitochondria and their subsequent transformation into AcCoA [28]. Etomoxir exhibited much stronger cytotoxicity in Plasmax that in a convenient medium (Figure 2f).

Another exception was bromopyruvate (BrPyr), which is often annotated as an inhibitor of hexokinase II (HKII) of the glycolysis pathway [29]. BrPyr suppressed proliferation of cells grown in Plasmax much more strongly than proliferation of cells in classical medium (Figure 2i). Since there is a significant difference in cytotoxicity of this compound and 2-deoxyglucose, another HK inhibitor, other possible targets for bromopyruvate were searched for in the literature. Indeed, this compound has previously been shown to affect other glycolytic enzymes and to suppress mitochondrial respiration through inhibition of succinate dehydrogenase of respiratory complex II [30]. To verify that the difference was due to redox pathway and not to glycolysis, activity of 2-deoxyglucose and BrPyr was examined in the absence and presence of N-acetylcysteine, one of the classical antioxidants. In line with our assumption, a 2-DG activity was resistant to the presence of antioxidant, whereas BrPyr in the presence of NAC completely lost its ability to suppress cell proliferation (Figure S2a,b), Similar changes in sensitivity to BrPyr were also observed for other cell lines (Figures S3 and S4). These results suggested that Plasmax affects redox pathways and, presumably, mitochondrial respiration.

Therefore, inhibitors of other respiratory complexes were evaluated as cytotoxic agents. No differences were found in the cytotoxic effects of rotenone, antimycin and sodium azide that inhibit respiratory complexes I, III and IV, respectively in the different media (Figure 2h,j,k). However, cells cultivated in Plasmax were more sensitive to oligomycin, an inhibitor of ATP synthase (Figure 2l). Other inhibitors of complex I such as metformin and phenphormin [31,32] were also equally toxic for cells in either media (Figure S2c,d).

**Figure 3:**
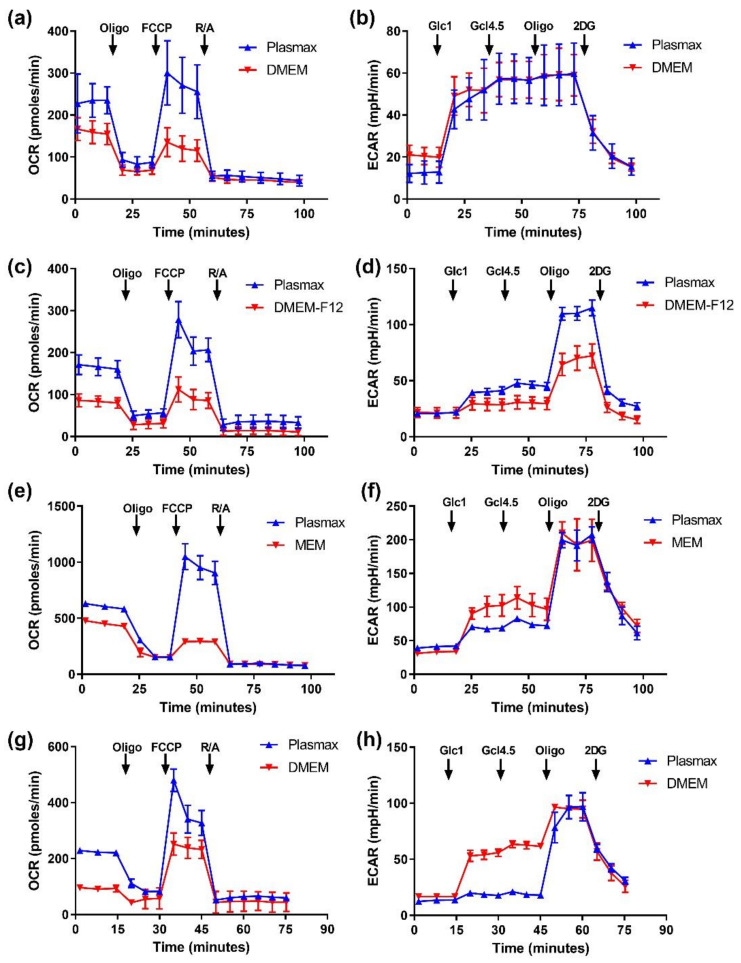
Cells, cultivated in Plasmax, exhibited elevated levels of basal mitochondrial respiration and spare respiratory capacity. Oxygen consumption rate (OCR) and extracellular acidification rate (ECAR) in Huh7.5 (**a**,**b**), A549 (**c**,**d**), HeLa (**e**,**f**) and Vero E6 cells (**g**,**h**) was measured in MitoStress (**a**,**c**,**e**,**g**) and GlycoStress (**b**,**d**,**f**,**h**) assays according to manufacturer’s instructions. In MitoStress oligomycin (Oligo, 1 µM), FCCP (0.45 µM), and a mixture of antimycin and rotenone (R/A, 1 µM each) were added, whereas in GlycoStress glucose was added to the final concentrations of 1 and 4.5 g/L (Glc1 and Glc4.5) followed by addition of oligomycin (Oligo, 1 µM), and 2-deoxyglucose (50 µM). Each experiment was performed three times, and typical graphs are presented.

Mitochondrial respiration as well as glycolysis in cells maintained in different media were assessed using a Seahorse analyzer. Maximum glycolytic activity was similar in either medium in case of all cells except A549, whereas some changes in basal glycolysis efficiencies were observed in this as well as HeLa and Vero E6 lines under two different glucose levels (Figure 3). However, the influence of Plasmax on basal glycolysis was in the opposite direction for these types of cell.

Strikingly, all cells maintained in Plasmax demonstrated markedly higher basal and maximum respiration levels (Figure 3). So substitution of classical media with Plasmax affects cell respiration with almost no effect on glycolysis.

### 3.3. Plasmax Decreases Lysosomal but Not Mitochondrial Mass

**Figure 4:**
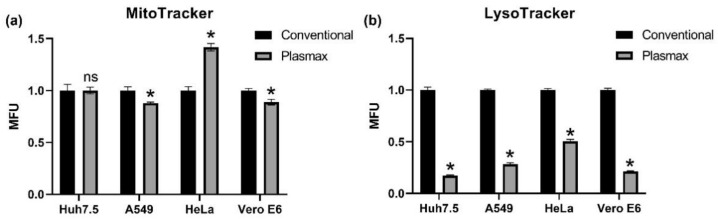
Plasmax medium decreases lysosomal mass in cells and has only marginal effect on mitochondrial mass. The cells were stained with Mitotracker Red CM-H2XROS (**a**) or LysoTracker Red DND-99 (**b**), and fluorescence levels were assessed by flow cytometry. Bars represent mean levels of fluorescence ± standard deviation. * *p* < 0.05 (two-tailed *t*-test, *n* = 3), ns—not significant.

The next step was to access if the changes in respiration were due to changes in mitochondrial mass. The latter was measured by two approaches: using mitochondria staining with Mitotracker Red with subsequent FACS analysis. In a second set of experiments, amounts of lysosomes in cells were assessed using Lysotracker. The results are presented in Figure 4. It can be clearly seen that substitution of classical medium with Plasmax when cultivating Huh7.5 and A549 cells had only a minor negative effect on mitochondrial mass (Figure 4a). However, the decrease in mitochondrial mass correlated with a small decrease in cell size (not shown). An increase in mitochondrial mass was shown only for HeLa cells (Figure 4). So the changes in respiration for the majority of cells results not from increased mitochondrial biogenesis but probably from a structural reorganization.

Interestingly, quantification of lysosomes using a fluorescent Lysotracker dye revealed that in all tested cell lines lysosomal mass in Plasmax was several times lower than in classical medium (Figure 4b). Further analysis on A549 cells showed that the change was not due to different concentration of glucose (1 vs. 4.5 g/L) or serum content (2.5 vs. 10%) between Plasmax and classical medium (Figure S5).

### 3.4. Plasmax Induces Assembly of Mitochondria into Network

**Figure 5:**
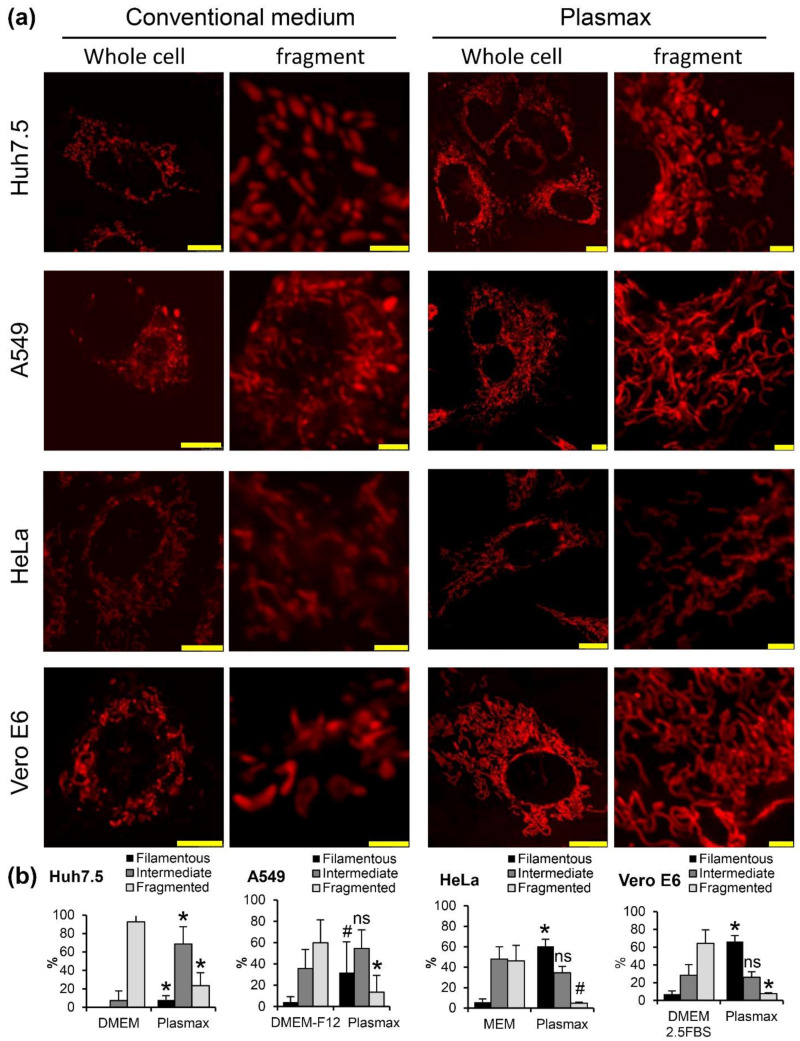
Plasmax medium induces formation of a mitochondrial network in cells. (**a**) The cells cultivated in convenient or Plasmax media were treated with MitoTracker Red, and staining was visualized by confocal microscopy. Bars denote 10 µm on the images with whole cells and 2.5 µm on the fragments. (**b**) Quantification of cells with filamentous, tubular or intermediate mitochondria from 2–5 independent experiments. Bars represent mean ± standard deviation. * *p* <0.05 compared to the respective bar in a convenient medium (two-tailed *t*-test, *n* = 5 for A549 cells and *n* = 3 for other cell lines), ^#^ 0.05 < *p* < 0.07), ns—not significant.

Another possible explanation of enhanced respiratory activity is a rearrangement of mitochondria into a vast network [33]. This was evaluated by confocal microscopy using all four cell lines seeded onto plates with glass bottom (in case of HeLa, coated with collagen) and stained with MitoTracked Red dye. For each cell line the experiments were repeated 3–5 times, and in each experiment 40–50 cells were visualized and their photos obtained. In each individual cell the mitochondrial staining was classified into fragmented mitochondria (>50% mitochondria fragmented), filamentous (<20) or intermediate. The typical images and quantitative analysis are presented on Figure 5a. One can see that in Plasmax, mitochondria tend to form networks, as is exemplified by transition from their fragmented state to intermediate in the case of Huh7.5 and HeLa cells, or from intermediate to filamentous in the case of A549 and Vero E6 lines (Figure 5b).

Mitochondrial turnover was evaluated in the HeLa cell line expressing Mitotimer protein that localizes to mitochondria and emits green light in case of “young” organelle and red light in case of “old” mitochondria [22]. Analysis by confocal microscopy showed that in all media mitochondria were of equal color (as can be seen for Plasmax medium in Figure S6a). FACS analysis confirmed that in both media the cells exhibited the same green/red ratio, although in Plasmax levels of MitoTimer expression were slightly higher than in MEM (Figure S6b). So Plasmax medium does not seem to change mitochondrial turnover.

### 3.5. RNA Viruses Can Replicate in Cells Grown in Plasmax Medium Albeit at Lower Levels

**Figure 6:**
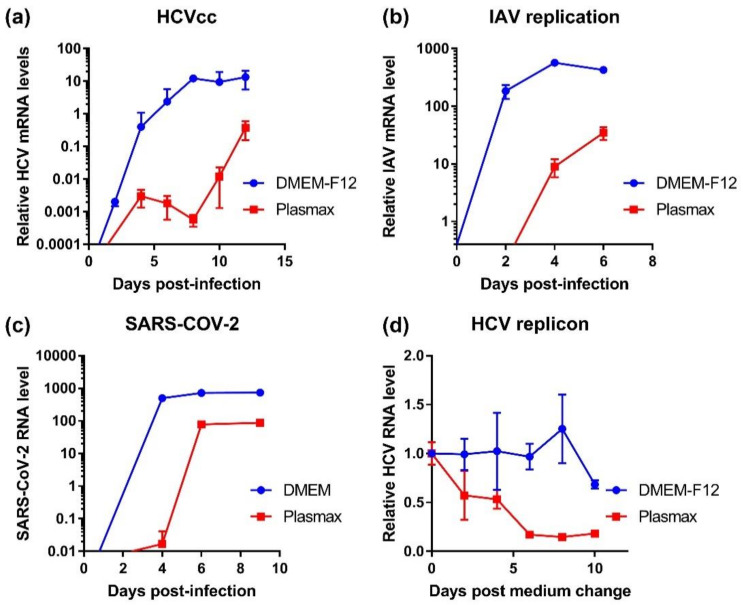
Cultivation of cells in Plasmax suppresses replication of various RNA viruses. Hh7.5 cells (**a**), A549 (**b**), or Vero E6 cells (**c**) were infected with hepatitis C virus, influenza A virus and SARS-CoV-2, respectively. Replication levels were assessed by quantification of viral RNA levels using RT-qPCR. For HCV as a non-cytopathogenic virus the levels were measured in cells and normalized to mRNA levels using GUS as a housekeeping gene, whereas for lytic IAV and SARS-CoV-2 levels of viral RNA were quantified in conditioned medium and normalized to volume units. (**d**) Huh7.5 cells harboring a bicistronic sub-genomic HCV replicon were cultivated in DMEM-F12 medium followed by change to Plasmax, and viral RNA levels were measured by RT-qPCR and normalized to GUS mRNA.

Next, we evaluated if Plasmax affects replication of various RNA viruses in the studied cell lines. The cells were adapted to Plasmax and infected with HCV (Huh7.5 cells), influenza A virus (A549), and SARS-CoV-2 (Vero E6) as well as enteroviruses (types 1 and 3 polioviruses, Coxsackie B5 virus), VSV-1, and Newcastle disease virus. Replication of HCV, IAV, and SARS-CoV-2 was monitored by RT-qPCR. As can be seen from Figure 6, replication of all these viruses was slower in cells in Plasmax that in classical medium and did not reach the maximum observed in standard media. Nevertheless, in all cases the cells supported viral replication. In the case of HCV, we evaluated whether the inhibition of virus reproduction was due to suppressed replication. Substitution of standard DMEM medium in Huh7.5 cells harboring a sub-genomic virus replicon showed that starting from day 2 levels of HCV RNA declined, and this decrease continued up to day 8.

Replication of other infections was assessed by measuring cytopathogenic effects. In case of NDV and type 3 poliovirus Plasmax slowed the replication and decreased its maximum levels both in HeLa and Vero E6 cells (Figure S7). At the same time, no such differences were found for type 1 poliovirus, Coxsackie virus and VSV-1. Therefore, Plasmax may not have any impact on rapidly replicating viruses.

### 3.6. Plasmax Medium Is Suitable for Study of Redox Biology of Viral Infections

**Figure 7:**
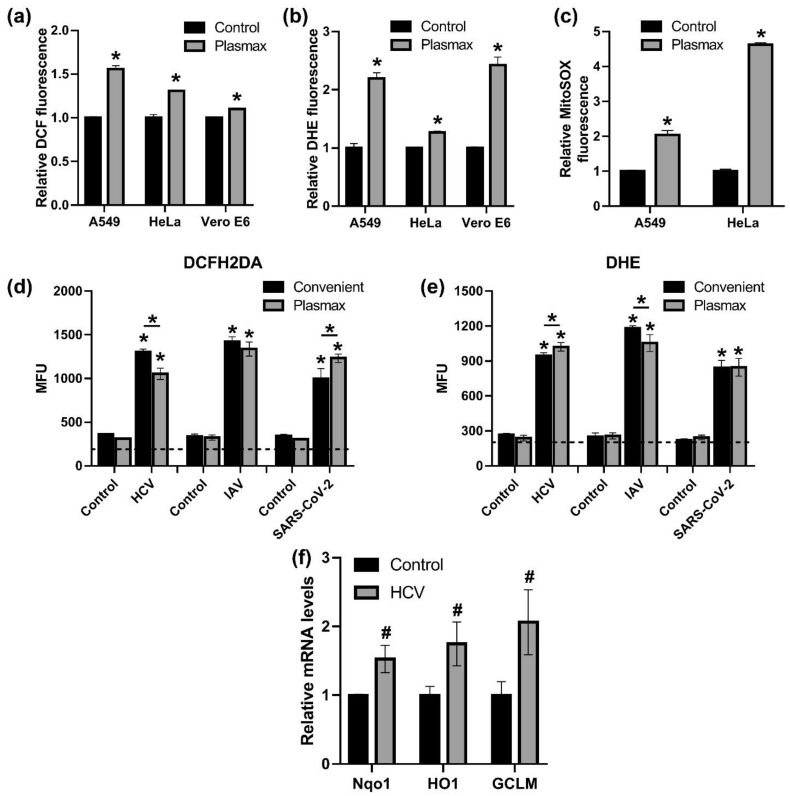
Plasmax medium is suitable for studies of redox biology of viral infections. (**a**–**c**) Levels of ROS production in cells cultivated in Plasmax or conventional medium were measured using 2′,7′-dichlorodehydrofluoresceine diacetate (DCFH2DA) (**a**), dihydroethidium (DHE) (**b**) or MitoSOX (**c**) by flow cytometry. (**d**,**e**) ROS production during the infections was accessed with DCFH2DA and DHE with measurement of fluorescence on a microplate reader. (**f**) Transcription of Nrf2-dependent genes in HCV-infected Huh7.5 cells cultivated in Plasmax was quantified by RT-qPCR, and the levels were normalized to the levels of GUS mRNA. Bars represent mean ± standard deviation. * *p* < 0.05 (two-tailed t-test on panels (**a**–**c**,**f**) or ANOVA with Tukey post-hoc test for panels (**d**,**e**); *n* = 3), ^#^ 0.05 < *p* < 0.07), ns—not significant.

Finally, we evaluated if Plasmax medium is suitable for redox biology studies. First, we evaluated levels of ROS production in three cell lines (A549, HeLa and VeroE6) by flow cytometry using a relatively non-specific DCFH2DA dye that reflects redox status in general, DHE that is oxidized by superoxide anion in cells in general and its mitochondrially-targeted MitoSOX derivative. All of them showed that cells in Plasmax exhibit higher levels of ROS production (Figure 7a–c). Next, production of ROS was quantified during HCV, IAV or SARS-CoV-2 infection. Since flow cytometry was not available in the BSL3 unit, levels of fluorescence were measured with a microplate reader. In the absence of infections the fluorescence was close to background (see dashed line on the Figure 7d,e) thus not allowing comparison of convenient medium and Plasmax. However, all three viruses strongly upregulated production of reactive oxygen species in both media.

Finally, since HCV is known to activate the antioxidant erythroid 2–related factor 2 (Nrf2) pathway that protects cells against oxidative and electrophilic stress [34], expression of three Nrf2-dependent genes was quantified in infected cells in Plasmax medium. Transcription of all three genes was upregulated (Figure 7f), demonstrating that Plasmax is suitable for redox biology studies.

## 4. Discussion

Introduction of Plasmax and HPLM into the metabolomics field represents a huge milestone towards development of adequate in vitro models for investigation of metabolic and redox pathways and modeling various diseases. However, due to their very recent development, these media have so far been used primarily by their inventors to characterize changes in specific pathways such as pyrimidine and serine biosynthesis, glutamine utilization, or HIF signaling. Cantor et al. demonstrated that urate in the medium suppresses pyrimidine nucleotide biosynthesis by inhibiting UMP synthase (UMPS) [18]. Normalization of arginine levels ensures conversion of this amino acid into non-proteinogenic ornithine and urea and not into arginino-succinate as in DMEM-F12 medium [19]. Reduced levels of pyruvate in such a physiological medium prevents artificial stabilization of HIF1 that occurs in convenient media. Biosynthesis of purine nucleotides in Plasmax occurs from hypoxanthine, present in the medium and absent in DMEM [35]. Plasmax also activates the de novo serine biosynthesis pathway [18,35] but with lower levels of its conversion into glutathione (GSH), glycine, purine nucleotides or thymidylate [35]. Furthermore, many other pathways have not yet been studied. Our paper expands this area by showing the impact of Plasmax on mitochondrial respiration and glycolysis.

The above discussed activation of the serine biosynthesis pathway, described by Vousden’s group, is mediated via ATF4 transcription factor of the integrated stress response [35]. The authors identified that ATF4 activation was due to lower levels of arginine in the medium (64 µM vs. 400 µM), as its supplement to the DMEM level blocked this activation. Initially we also observed activation of ATF4 targets such as PSPH, PHGDH and PSAT1 from the serine biosynthesis pathway or ASNS that converts aspartate to asparagine [25]. However, this effect disappeared when culture medium was replaced every 24 h pointing to nutrient exhaustion. ATF4 is known to be activated during inhibition of cap-dependent translation via phosphorylation of eukaryotic initiation factor 2a (eIF2a) by four different protein kinases [26]. They include double-stranded RNA-activated protein kinase (PKR), PRK-like protein kinase (PERK) of unfolded protein response, HRI, and GCN2. The latter is activated during exhaustion of the amino acid pool through binding of uncharged tRNAs [36]. At the same time exhaustion of two amino acids, leucine and arginine, was shown previously to mediate Gcn2-dependent activation of mTORC1, although in an ATF4-independent manner [37]. We did not evaluate the role of GCN2 in activation of serine biosynthesis, but its role in induction of de novo serine biosynthesis pathway upon prolonged incubation of cells in Plasmax without medium change cannot be excluded. To sum up, cultivation of cells in Plasmax requires more frequent medium change.

We showed here that Plasmax ensures more efficient mitochondrial respiration in every cell line studied. At the same time, the initial paper does not mention such changes [19]. This discrepancy could result from different periods of cell maintenance in Plasmax (7 days in our study vs. 2 days in [19]). This falls in line with an observation that changes in cell morphology, especially clear in Huh7.5 cells, become visible only after 72 h after substitution of DMEM with Plasmax. Similarly, decrease in HCV replication levels in Huh7.5 cells harboring a replicon start two days after medium change and develop during a week. Both facts indicate that adaptation to Plasmax is slow, being another caveat during our experiments.

Increased respiration was accompanied not by an increase in mitochondrial mass but by rearrangement of the mitochondrial network. Indeed, it has been reported that filamentous mitochondria display enhanced oxidative phosphorylation compared to the fragmented ones [33,38]. First, it could be speculated that Plasmax affects fusion/fission process which remained out of scope of the present study. If so, future endeavors are needed to unveil the interplay between various metabolites and fusion/fission. Second, respiration could be affected by assembly of respiratory complexes into “supercomplexes” (SCs) that are characterized by enhanced efficiency of electron flow and ATP production [39]. The most known regulator of CSs formation is SCAF1/COX7A2L [40,41], but its transcription was unaffected by Plasmax (data not shown) arguing against the effect of the medium on SC arrangement. Third, increased levels of respiration could be merely due to increased levels of substrates provided to the TCA cycle by glycolysis, glutaminolysis, fatty acid oxidation or other pathways. The first two can be excluded, as inhibitors of hexokinase (2DG) and glutaminase (GLS1) showed similar toxicity towards cells maintained in classical media and Plasmax. In contrast, etomoxir that disrupts fatty acid oxidation from TCA cycle [28,42] was more toxic to cells in Plasmax that in other media. This indicates that cells in Plasmax are more dependent on fatty acid oxidation pathway. Fatty acids are provided by serum added to the culture medium. Plasmax contains four-fold lower serum levels than classical media for majority of cell lines (2.5 vs. 10%) except for Vero E6 cells (2.5%). However, in all cases cells in Plasmax exhibited higher levels of respiration pointing to the medium components as regulators of the process. It could be speculated that the key metabolite is carnitine that is absent in classical media and present in Plasmax. As it functions as shuttling molecule required for import of acyl groups produced by FA oxidation into mitochondria for AcCoA production [28], it could merely support higher input of the pathway to TCA cycle. Another metabolite that could have contributed to enhanced respiration could be proline that is present in Plasmax at higher levels than in DMEM. Proline can be converted into glutamate via pyrrolidine-5-carboxylate [43]. However, this hypothesis requires verification by flux analysis using mass-spectrometry, which is beyond the scope of our study.

One of the intriguing findings in this report was a markedly reduced number/mass of lysosomes in cells in Plasmax compared to classical media. We did not investigate the mechanisms underlying these changes; additional studies are needed. But it is already clear that use of Plasmax instead of DMEM/MEM/F12 can affect results of studies of lysosome-associated processes such as autophagy and mitophagy, lysosome-associated protein processing and degradation as well as antigen presentation, cholesterol transport, mTORC1 and AMPK signaling and many others [44,45]. A lysosomal SLC38A9 transporter is an arginine-sensing protein critical for mTORC1 activation, so changes in lysosomal mass may underlie induction of serine biosynthesis discussed above [46]. Lysosomes were also shown to store cystine [47], accumulation of which induces mitochondria depolarization [48] and is highly likely to have decreased oxidative phosphorylation. If so, a decrease in lysosome mass can ensure proper functioning of mitochondria. Again, Plasmax may allow studies of the role of lysosomes in amino acid metabolism.

Our study demonstrates that Plasmax or presumably HPLM can be used for propagation of RNA viruses and investigation of their molecular biology. The mechanisms of decreased replication in Plasmax are not quite clear. Using the HCV replicon system, we showed that the effect was not due to differences in glucose or FBS levels, nor to presence/absence of lactate and selenite (data not shown). Probably, this decrease is due to activated oxidative phosphorylation, observed in all these cases. However, the effect is probably indirect, as replication of these viruses occurs outside mitochondria: at the endoplasmic reticulum (HCV) [49], in the nucleus (IAV) [50], or the cytoplasm (enteroviruses) [51] but, of course, rearrangement of mitochondria into networks may affect the cytoskeleton [52], and the reduced number of lysosomes are likely to effect various processes including endo- and exocytosis, pH state and several signaling pathways [44,45]. Search for the underlying molecular mechanism may lead to identification of cellular factors that are required for efficient replication of a wide spectrum of viruses.

On the one hand, reduced replication of various viruses in Plasmax raises the question as to whether Plasmax can mitigate any virus-induced changes. So far, we have examined expression of the genes controlled by the Nrf2 factor, as the virus and several of its proteins have previously been shown to activate the Nrf2/ARE pathway [16,53]. RT-qPCR analysis revealed that the virus induces Nqo1, HO-1 and GCLM, the Nrf2-dependent genes in Plasmax, despite reduced viral replication levels. This might be explained by lower levels of pyruvate and presence of lactate in Plasmax that shift the NAD/NADH ratio, and therefore redox balance, from an artificially reduced state to a more physiological state. On the other hand, we should remember that for many viruses highly permissive cell lines and sometimes strains are used. For example, the HCVcc model is based on one of the few artificial hepatoma cell lines that support HCV replication at extremely high levels due to defect in interferon signaling [54,55], and on a unique JFH1 virus strain from a patient with fulminant hepatitis [56,57]. Similarly, Vero E6 are known to provide the highest levels of SARS-CoV-2 production compared to other lines [58]. So usage of Plasmax may lower down virus replication towards more physiological levels. All this indicates that Plasmax is not only suitable for molecular virology studies but may also be useful for the redox biology community. Of course, it will also be used in future to assess virus-induced metabolic changes.

Oxidative stress contributes to the development of various pathologies associated with viral respiratory infections, hepatitis B and C and human immunodeficiency viruses [59,60,61]. For SARS-CoV-2 it was noted that the levels of stress markers such as 4-hydroxyhonenal (HNE) are much higher in critically ill patients who died from COVID-19 than in survivors [62]. Our study clearly shows that not only the HCV and influenza virus but also SARS-CoV-2 trigger massive production of reactive oxygen species. This falls in line with the observations from other coronaviruses such as SARS-CoV, as well as other respiratory viral infections [61]. Other groups reported that SARS-CoV-2 Spike protein up-regulates ROS production in macrophages and endothelial cells upon extracellular treatment [63,64]. A similar effect was described for monocytes, HEK293-ACE and Vero E6 cells infected with the virus or Spike-bearing lentivirus [65,66]. In our study the “net” levels of ROS production were similar between conventional and Plasmax media. However, taking into account much lower levels of infections in Plasmax, the relative prooxidant effect of either virus is much stronger in Plasmax, so usage of this medium for investigation of mechanisms by which SARS-CoV-2 as well as other viruses affect cellular redox pathways is warranted.

## 5. Conclusions

To sum up the data, Plasmax medium changes the metabolism of various cell lines by increasing their respiration, likely due to the rearrangement of mitochondria into networks, and decreases lysosomal content. These changes lead to slower replication of various RNA viruses, albeit not all, and lower levels of their RNA. Nevertheless, the medium is suitable for studies of their redox biology and virus-associated metabolic reprogramming.

## Data Availability

The data presented in this study are available in article and Supplementary Material.

## Supplementary Materials

The following are available online at https://www.mdpi.com/article/10.3390/antiox11010097/s1, Figure S1: Regular medium change prevents up-regulation of transcription of ATF4-dependent genes of asparagine and serine biosynthesis, Figure S2. Cytotoxicity of glycolysis and respiration inhibitors in HeLa cells in Plasmax or MEM, Figure S3. Cultivation of Huh7.5 cells in Plasmax leads to their enhanced sensitivity to inhibitors of fatty acid catabolism, mitochondrial respiratory complexes, and ferroptosis inducer, Figure S4. Cultivation of A549 cells in Plasmax leads to their enhanced sensitivity to inhibitors of fatty acid catabolism and mitochondrial complex II, Figure S5. Decrease in lysosomal mass in Plasmax does not result from different levels of fetal bovine serum (FBS) or glucose, Figure S6. Plasmax does not affect mitochondrial turnover, Figure S7. Impact of Plasmax medium on replication of several RNA viruses in HeLa or Vero E6 cells, Table S1. Components and concentrations of Plasmax, Table S2. Primers used for real-time PCR analysis.
