# Supplementary material for: Cultivation of Cells in a Physiological Plasmax Medium Increases Mitochondrial Respiratory Capacity and Reduces Replication Levels of RNA Viruses
Source: Antioxidants (Basel). 2021 Dec 30;11(1):97. doi: 10.3390/antiox11010097 (PMC8772912; doi:10.3390/antiox11010097)
Supplement: Supplementary file 1 [file antioxidants-11-00097-s001.zip › antioxidants-1512045-Supplementary V3 -2nd proof-AI.pdf]

Supplementary material

Cultivation of cells in a Physiological Plasmax medium increases mitochondrial respiratory capacity and reduces replication levels of RNA viruses

Michail V. Golikov [1], Inna L. Karpenko [1], Anastasiya V. Lipatova [1], Olga N. Ivanova [1], Irina T. Fedyakina [2], Viktor P. Larichev [2], Natalia F. Zakirova [1], Olga G. Leonova [1], Vladimir I. Popenko [1], Birke Bartosch [3], Sergey N. Kochetkov [1], Olga A. Smirnova [1] and Alexander V. Ivanov [1,*]

[1] Engelhardt Institute of Molecular Biology, Russian Academy of Sciences, Moscow, 119991, Russia; cool.mik3492594@yandex.ru (M.G.); ilkzkil@gmail.com (I.K.); lipatovaanv@gmail.com (A.L.); nat_zakirova@mail.ru (N.Z.); olgaum@yandex.ru (O.I.); varjag@aport2000.ru (O.L.); popenko@eimb.ru (V.P.); snk1952@gmail.com (S.K.); aivanov@yandex.ru (A.I.)

[2] Gamaleya National Research Centre for Epidemiology and Microbiology of the Ministry of Russia, Moscow, Russia; irfed2@mail.ru (I.F.); vlaritchev@mail.ru (V.L.)

[2] Univ Lyon, Université Claude Bernard Lyon 1, INSERM 1052, CNRS 5286, Centre Léon Bérard, Centre de recherche en cancérologie de Lyon, Lyon, 69434, France; birke.bartosch@inserm.fr

* Correspondence: aivanov@yandex.ru or aivanov@eimb.ru; Tel.: +74991356065

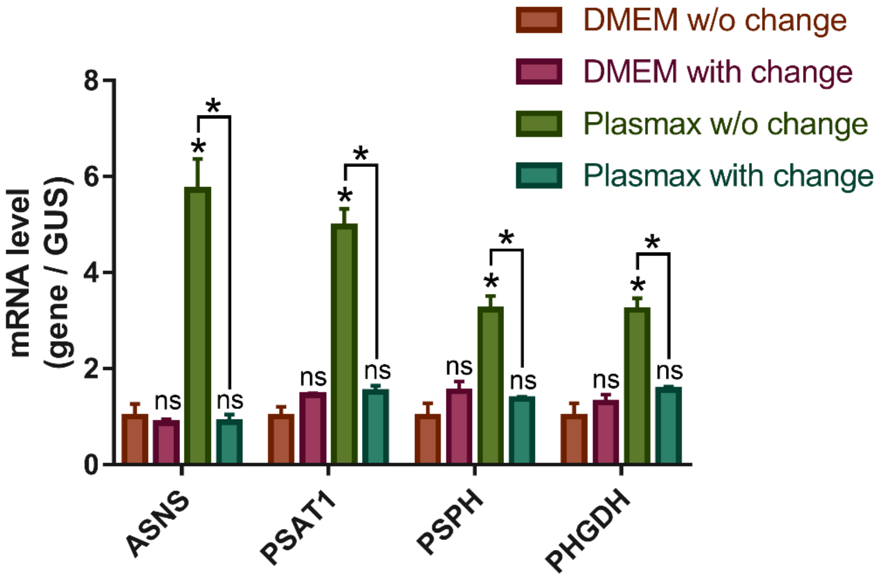

Figure S1. Regular medium change prevents up-regulation of transcription of ATF4-dependent genes of asparagine and serine biosynthesis. The Huh7.5 cells were cultivated in DMEM or Plasmax for three days without or with changing medium every 24 h. Bars represent mean ± standard deviation. *p<0.05 compared to DMEM w/o medium change if not stated otherwise (ANOVA with Tukey post-hoc-test, n=3), ns – not significant.

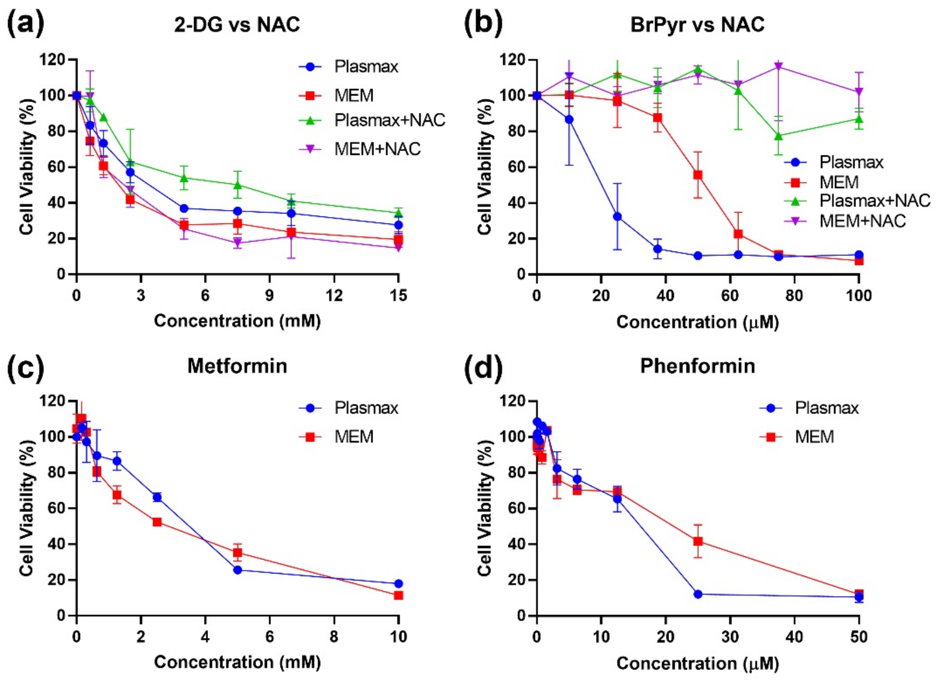

Figure S2. Cytotoxicity of glycolysis and respiration inhibitors in HeLa cells in Plasmax or MEM. The HeLa cells cultivated in Plasmax or MEM as a conventional medium were treated with 2-deoxyglucose (2-DG) (**a**) or bromopyruvate (**b**) in the absence or presence of 1 mM N-acetylcysteine, with metformin (**c**) or phenformin (**d**) for 72 h, and cell viability was accessed my conventional MTT test. The values were normalized to the untreated cells.

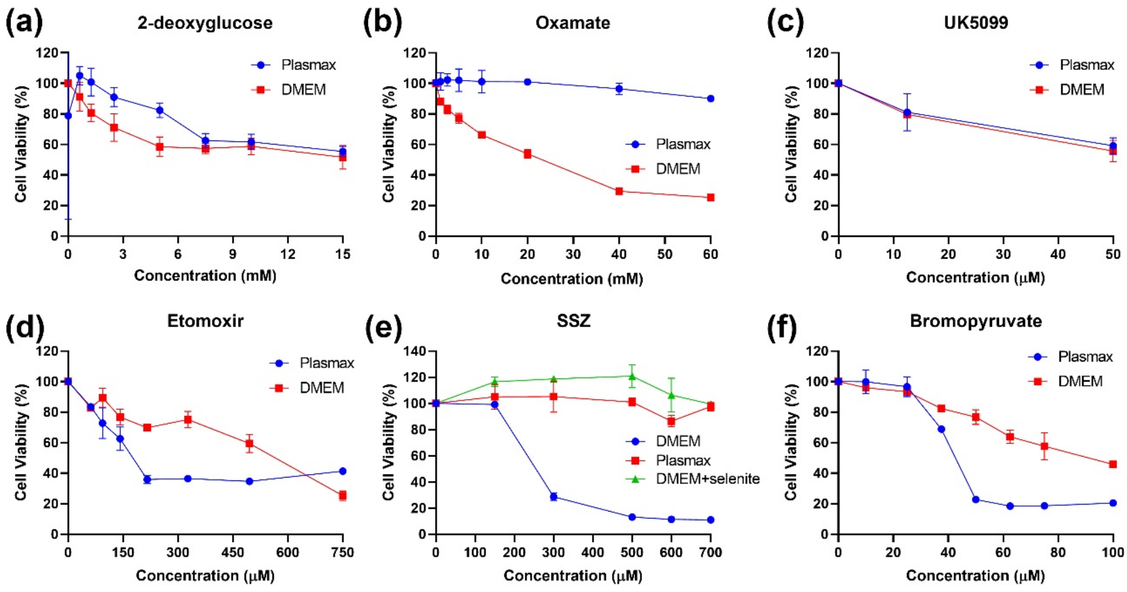

Figure S3. Cultivation of Huh7.5 cells in Plasmax leads to their enhanced sensitivity to inhibitors of fatty acid catabolism, mitochondrial respiratory complexes, and ferroptosis inducer. The Huh7.5 cells cultivated in Plasmax or DMEM as a conventional medium were treated with inhibitors of glycolysis (2-deoxyglucose and oxamate—**a**,**b**), mitochondrial pyruvate transporter (UK5099—**c**), fatty acid degradation (etomoxir—**d**), cysteine/glutamate antiporter (sulfasalazine, SSZ—**e**), or of respiratory complex II (bromopyruvate—**f**) for 72 h, and cell viability was accessed my conventional MTT test. The values were normalized to the untreated cells.

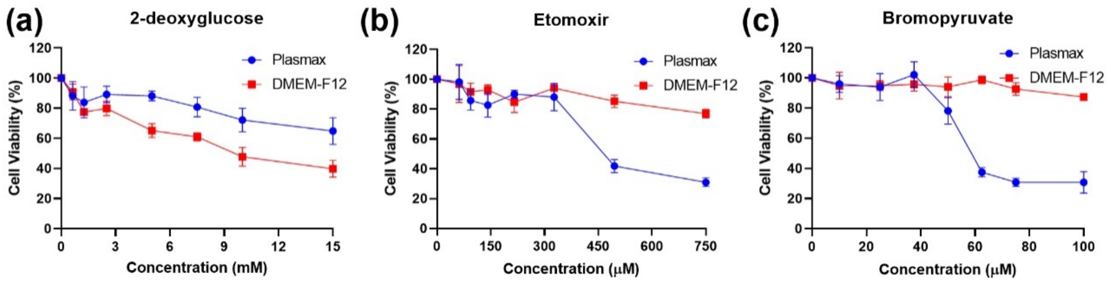

Figure S4. Cultivation of A549 cells in Plasmax leads to their enhanced sensitivity to inhibitors of fatty acid catabolism and mitochondrial complex II. The A549 cells cultivated in Plasmax or DMEM-F12 as a conventional medium were treated with inhibitors of glycolysis (2-deoxyglucose—**a**), fatty acid degradation (etomoxir—**b**), or respiratory complex II (bromopyruvate—**c**) for 72 h, and cell viability was accessed my conventional MTT test. The values were normalized to the untreated cells.

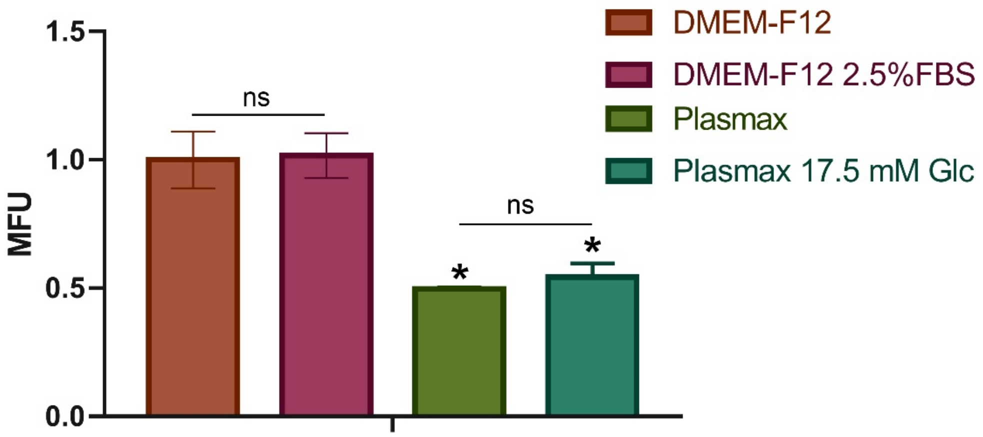

Figure S5. Decrease in lysosomal mass in Plasmax does not result from different levels of fetal bovine serum (FBS) or glucose. A549 cells were cultivated in DMEM-12 medium with standard (10%) or reduced (2.5%) FBS, or in Plasmax with standard (5 mM) or increased (17.5 mM) glucose. Then they were stained with LysoTracker Red DND-99, and fluorescence levels were accessed by flow cytometry. Bars represent mean levels of fluorescence ± standard deviation. *$p<0.05$ compared to DMEM-F12 change if not stated otherwise (ANOVA with Tukey post-hoc-test, n=3), ns – not significant.

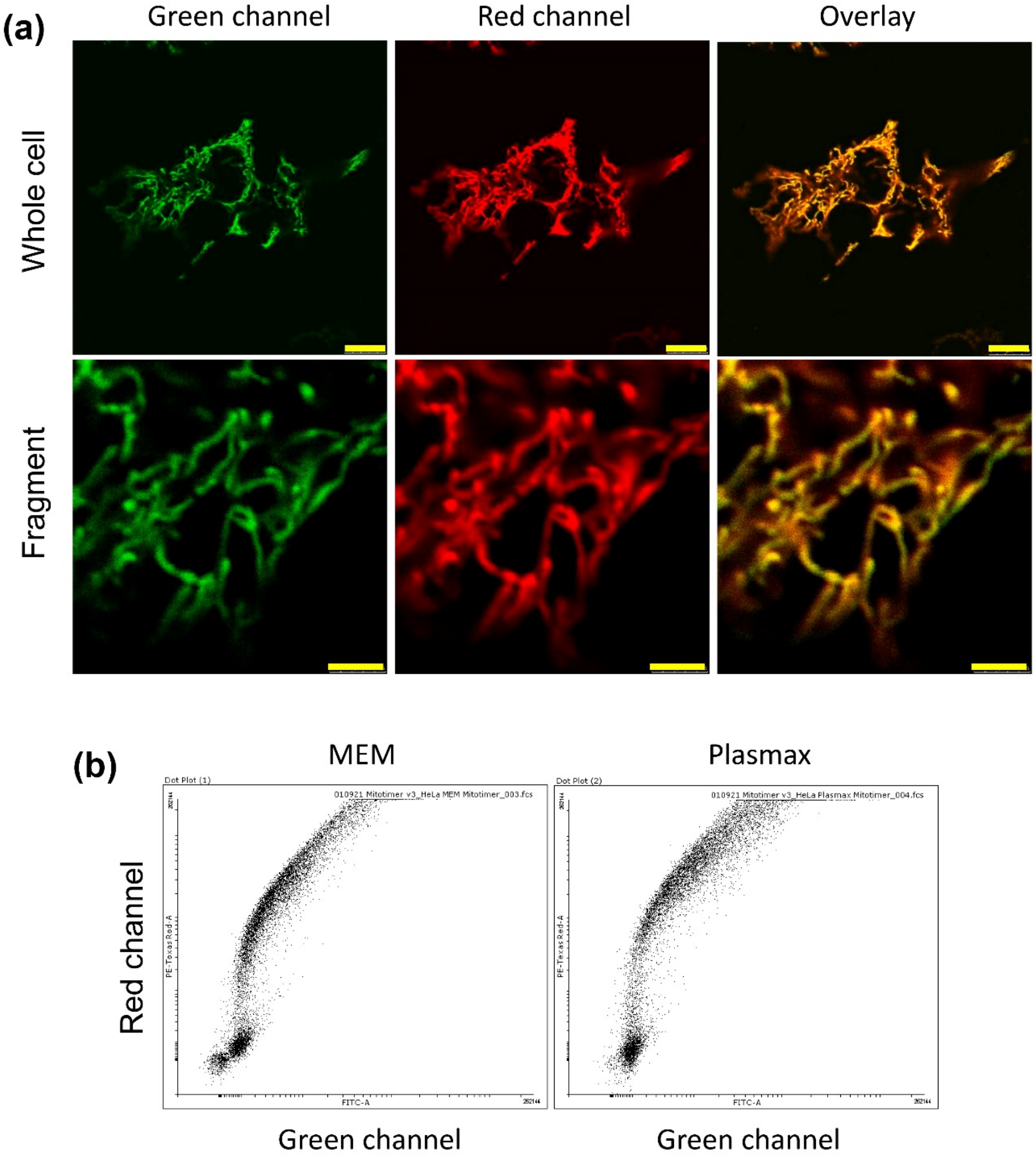

Figure S6. Plasmax does not affect mitochondrial turnover. HeLa cells expressing MitoTimer protein were analyzed by confocal microscopy (**a**) or FACS analysis (**b**). Bars denote 10 µm on the images with whole cells and 2.5 µm on the fragments.

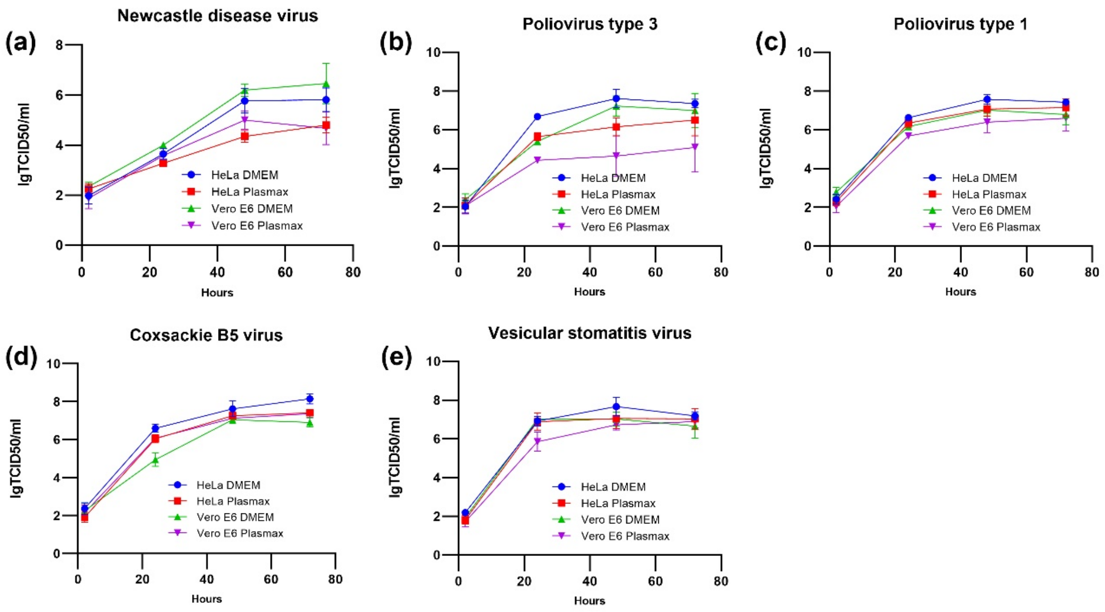

Figure S7. Impact of Plasmax medium on replication of several RNA viruses in HeLa or Vero E6 cells. (**a**), Newcastle disease virus (H2 strain) (**b**), poliovirus type 3 (Sabin) (**c**) poliovirus type 1 (Sabin), (**d**) Coxsackie virus B5 (LEV15 strain) (**e**) vesicular stomatitis virus (Indiana strain). Replication levels were assessed by Reed and Meunch method, accomplished on appropriate sensitive cell cultures (enteroviruses were propagated in RD cells (ATCC CCL-136), VSV-I in BHK21 cells (ATCC C-13), NDV-H2 in Vero cells (ATCC CCL-81).

Table S1. Components and concentrations of Plasmax.

Component	Concentration (μM)	Cat #	Manufacturer
L-Alanine	510	05129-25G	Sigma-Aldrich
L-Arginine	64	A6969-100G	Sigma-Aldrich
L-Asparagine	41	69102070	Reanal
L-Aspartic acid	6	A7219-100G	Sigma-Aldrich
L-Glutamate	98	49621-250G	Sigma-Aldrich
Glycine	330	G5417-100G	Sigma-Aldrich
L-Histidine	120	H5659-25G	Sigma-Aldrich
L-Isoleycine	140	I2752-25G	Sigma-Aldrich
L-Leycine	170	L8912-25G	Sigma-Aldrich
L-Lysine	220	L8662-100G	Sigma-Aldrich
L-Methionine	30	M5308-25G	Sigma-Aldrich
L-Phenylalanine	68	P5482-25G	Sigma-Aldrich
L-Proline	360	P5607-25G	Sigma-Aldrich
L-Serine	140	S4311-25G	Sigma-Aldrich
L-Threonine	240	89179-10G	Sigma-Aldrich
L-Tryptophan	78	93669-10G	Sigma-Aldrich
L-Tyrosine	74	T8566-25G	Sigma-Aldrich
L-Valine	230	V0513-25G	Sigma-Aldrich
L-Citrulline	55	BP375-25	Biotech-Fisher
L-Cystine	65	C7602-25G	Sigma-Aldrich
L-Ornitine	80	02375-25G	Sigma-Aldrich
α-Aminobutyrate	41	162663-25G	Sigma-Aldrich
L-Homocysteine	9	69453-10MG	Sigma-Aldrich
4-Hydroxy-L-proline	13	56250-5G	Sigma-Aldrich
L-Pyroglutamate	20	P5960-25G	Sigma-Aldrich
N-Acetylglycine	70	A16300-100G	Sigma-Aldrich
L-Carnosine	6	C9625-10MG	Sigma-Aldrich
GSH	37	G4251-25G	Sigma-Aldrich
Taurine	130	T8691-25G	Sigma-Aldrich
Betaine	72	B2629-50G	Sigma-Aldrich
Acetate	42	S-8625	Sigma-Aldrich
Acetone	55	650501	Sigma-Aldrich
Acetyl carnitine	5	A6706-1G	Sigma-Aldrich
Citrate	114	C-8532	Sigma-Aldrich
Carnitine	46	C0158-1G	Sigma-Aldrich
Creatine	37	C3630-25G	Sigma-Aldrich
Creatinine	74	C4255-10G	Sigma-Aldrich
Formate	33	168610050	Acros Organics
Glycerol	82	G5516-1L	Sigma-Aldrich
2-Hydroxybutyrate	31	220116-5G	Sigma-Aldrich
3-Hydroxybutyrate	77	298360-1G	Sigma-Aldrich
3-Hydroxyisobutyrate	20	16842-100MG	Sigma-Aldrich

Hypoxanthine	5	H9477-1G	Sigma-Aldrich
Lactate	500	71718-10g	Aldrich
Methyl acetoacetate	41	537354-100G	Sigma-Aldrich
Succinate	23	S9637-100G	Sigma-Aldrich
Uracil	2	U1128-25G	Sigma-Aldrich
Urea	3000	U5378-100G	Sigma-Aldrich
Uridine	3	43750	Sigma-Aldrich
L-Cysteine	33	C7477-25G	Sigma-Aldrich
Ammonium Chloride	50	10317	Reachem
Cupric Sulfate	0.0052	197730010	Acros Organics
Ferric Nitrate	0.1238	F8508-100G	Sigma-Aldrich
Ferric Sulfate	1.0428	F8633-250G	Sigma-Aldrich
Zinc Sulfate	1.5	Z4750-100G	Sigma-Aldrich
Sodium Selenite	0.0289	F080	Paneco
Ammonium Metavanadate	0.0026	398128-50G	Sigma-Aldrich
Manganous Chloride	0.0002	271412500	Acros Organics
Urate	270	U2875-5G	Sigma-Aldrich
D-Biotin	4.1	B6891-100ML	BME vitamin mix from Sigma-Aldrich
Choline	7.1		
Folate	2.3		
myo-Inositol	11.1		
Niacinamide	8.2		
D-Pantothenic acid hemicalcium	4.2		
Pyridoxine	4.9		
Riboflavin	0.3		
Thiamine	3		
L-Glutamine	650	49419-25G	Sigma-Aldrich
Vitamin B12	0.005	V6629-100MG	Sigma-Aldrich
Ascorbate	62	A4034-100G	Sigma-Aldrich
Pyruvate	100	P5280-25G	Sigma-Aldrich
Calcium Chloride	1800	24010043	Earle's Balanced Salt Solution (EBSS) from Thermo
Magnesium Sulfate	813		
Potassium Chloride	5330		
Sodium Chloride	118706		
Sodium Phosphate	1010		
D-Glucose	5560		
Phenol Red	25		
Sodium Bicarbonate	26191		

Table S2. Primers used for real-time PCR analysis.

Gene/virus	Forward	Reverse	Genbank mRNA accession number
GUS	5'-CGTGGTTGGAGAGCTCATTTGGAA-3'	5'-ATTCCCCAGCACTCTCGTCGGT-3'	NM_000181.4
HCV	5'-GTCTAGCCATGGCGTTAGTA-3'	5'-CTCCCGGGGCACTCGCAAGC-3'	Non applicable
IAV	5'-CTCTTTGATCTTCCACAAGRGGT-3'	5'-GCCGCTGTTACCCTATCCAA-3'	Non applicable
SARS-CoV-2	5'-ATGCTGCAATCGTGCTACAA-3'	5'-GACTGCCGCCTCTGCTC-3'	Non applicable
PSAT	5'-TGCCGCACTCAGTGTTGTTA-3'	5'-GCTAGCAATTCCCGCACAAG-3'	NM_058179.4
PSPH	5'-CACGGTCATCAGAGAAGAAG-3'	5'-GGTTGCTCTGCTATGAGTCT-3'	NM_001370518.1
PHGDH	5'-CACATTCTTGGGCTGAAC-3'	5'-TTATTAGACGGTTATTGCTGTA-3'	NM_006623.4
ASNS	5'-TGCACGCCCTCTATGACAAT-3'	5'-CACCTTTCTAGCAGCCAGTA-3'	NM_133436.3
CHOP	5'-AGAACCAGGAAACGGAAACAGA-3'	5'-TCTCCTTCATGCGCTGCTTT-3'	NM_001195053.1
ATF4	5'-CCAACAACAGCAAGGAGGAT-3'	5'-GTGTCATCCAACGTGGTCAG-3'	NM_001675.4
Nqo1	5'-CCGTGGATCCCTTGCAGAGA-3'	5'-AGGACCCTTCCGGAGTAAGA-3'	NM_000903.3
HO-1	5'-CCAGCAACAAAGTGCAAGATTC-3'	5'-TCACATGGCATAAAGCCCTACAG-3'	NM_002133.3
GCLC	5'-GGATTTGGAAATGGGCAATTG-3'	5'-CTCAGATATACTGCAGGCTTGGAA-3'	NM_001498.4
GCLM	5'-TGCAGTTGACATGGCCTGTT-3'	5'-TCACAGAATCCAGCTGTGCAA-3'	NM_002061.4
